# Reduced Sensory-Evoked Locus Coeruleus-Norepinephrine Neural Activity in Female Rats With a History of Dietary-Induced Binge Eating

**DOI:** 10.3389/fpsyg.2019.01966

**Published:** 2019-09-04

**Authors:** Nicholas T. Bello, Chung-Yang Yeh, Morgan H. James

**Affiliations:** ^1^Department of Animal Sciences, School of Environmental and Biological Sciences, Rutgers, The State University of New Jersey, New Brunswick, NJ, United States; ^2^Rutgers Brain Health Institute, Rutgers Biomedical and Health Sciences, Rutgers University, New Brunswick, NJ, United States; ^3^Florey Institute for Neuroscience and Mental Health, Parkville, VIC, Australia

**Keywords:** stress axis, eating pathologies, binge eating disorder, bulimia, estrus cycle, locus coeruleus

## Abstract

Noradrenergic pathways have been implicated in eating pathologies. These experiments sought to examine how dietary-induced binge eating influences the neuronal activity of the locus coeruleus (LC)-norepinephrine (NE) system. Young adult female Sprague Dawley rats (7–8 weeks old) were exposed to a repeated intermittent (twice weekly) cycle of 30-min access to a highly palatable sweetened fat (i.e., vegetable shortening with 10% sucrose) with or without intermittent (24 h) calorie restriction (Restrict Binge or Binge groups, respectively). Age- and weight-matched female control rats were exposed to standard chow feeding (Naive group) or intermittent chow feeding (Restrict group). The Binge and Restrict Binge groups demonstrated an escalation in sweet-fat food intake after 2.5 weeks. On week 3, *in vivo* single-unit LC electrophysiological activity was recorded under isoflurane anesthesia. Restrict Binge (20 cells from six rats) and Binge (27 cells from six rats) had significantly reduced (approximate 20% and 26%, respectively) evoked LC discharge rates compared with naive rats (22 cells, seven rats). Spontaneous and tonic discharge rates were not different among the groups. Signal-to-noise ratio was reduced in the groups with intermittent sweetened fat exposure. In order to investigate the neuropeptide alterations as a consequence of dietary binge eating, relative gene expression of neuropeptide Y (NPY), glucagon-like peptide 1 receptor (GLP-1r), prodynorphin, and related genes were measured in LC and hypothalamic arcuate (Arc) regions. *Glp-1r*, *Npy2r*, and *Pdyn* in LC region were reduced with repeated intermittent restriction. *Npy1r* was reduced by approximately 27% in ARC of Restrict compared with Naive group. Such data indicate that dietary-induced binge eating alters the neural response of LC neurons to sensory stimuli and dampens the neural stress response.

## Introduction

Binge eating is an eating pathology characterized by a sense of a “loss of control” over eating accompanied by recurrent overeating episodes (American Psychiatric Association, 2013). While the triggers for binge eating episodes vary in susceptible individuals, perceived stress or stress coping is often reportedly associated with binge eating (Pendleton et al., 2001; Rikani et al., 2013; Rospond et al., 2015). Norepinephrine (NE) is a classic hormone and neurotransmitter traditionally implicated in stress reactivity (Euler, 1948). Early studies by Kaye and colleagues demonstrated that central NE were lower in women with bulimia nervosa (BN) and central NE fluctuations were related to bingeing and vomiting behaviors (Kaye et al., 1990a,b). In addition, selective NE reuptake inhibitors, atomoxetine and reboxetine, have been shown to be effective in the short-term management of binge eating disorder (BED) and BN (El-Giamal et al., 2000; Fassino et al., 2004; Silveira et al., 2005; McElroy et al., 2007). Despite this, the central noradrenergic pathways on binge eating behaviors have not been well-characterized. In addition, the central noradrenergic pathways have been implicated in other physiological processes not directly related to stress control, such as attention and nociception, that could influence eating pathologies (Conti et al., 1997; Aston-Jones et al., 2000; Berridge and Waterhouse, 2003; Ramos and Arnsten, 2007; Valentino and Van Bockstaele, 2008; Taylor and Westlund, 2017; Hayashida and Eisenach, 2018).

Central noradrenergic pathways are clustered into seven cell groups (A1–A7); however, a majority of the forebrain NE originates from the A6 region, locus coeruleus (LC) (Sawchenko and Swanson, 1982). Indeed, the LC is a pontine brainstem nucleus that is the principal source of NE in the cortex, hippocampus, and cerebellum (Foote et al., 1983; Myers and Rinaman, 2002). The activity of LC-NE neurons is modulated, in a large part, by intrinsic and afferent peptidergic input (Horvath et al., 1999; Valentino and Van Bockstaele, 2008). One known neuropeptide modulator of LC-NE function is neuropeptide Y (NPY) (Kim et al., 1998; Britton et al., 2000).

NPY is a 36 amino acid peptide and is the most abundant peptide in the CNS (Tatemoto et al., 1982; Adrian et al., 1983; Allen et al., 1983; Clark et al., 1984). NPY is also distributed in several distinct neural structures along the neuraxis (Adrian et al., 1983; Allen et al., 1983). Several lines of research indicate that NPY is a potent orexigenic peptide released by a population of cells in the arcuate nucleus (Arc) of the hypothalamus (Beck et al., 1990a,b). Not only is NPY a potent stimulator of food intake, but NPY has anxiolytic effects in extrahypothalamic areas (Kask et al., 1998; Palmiter et al., 1998). The LC has a dense population of NPY receptors (Y1, Y2, and Y5) and NPY is co-expressed in 20–40% of LC-NE neurons (Everitt et al., 1984; Holets et al., 1988; Smialowska, 1988; Wolak et al., 2003; Stanic et al., 2006; Theisen et al., 2018). Related to the overlap of feeding behaviors and stress resilience, one notion is that there is an interaction between Arc and LC NPY systems. Specifically, overexpression of Arc NPY not only reduces brown adipose tissue and the associated metabolic alterations but also decreases LC levels of tyrosine hydroxylase (TH), a critical enzyme for the synthesis of NE (Shi et al., 2013). Other experimental paradigms have demonstrated a further link between alterations in Arc-NPY expression and LC-NE activity (Baker et al., 1996; Makino et al., 2000). In particular, long-term treatment (>14 days) with an antidepressant, imipramine, reduces Arc-NPY and reduces LC-TH expression (Baker et al., 1996). In addition, a small number (~2%) of Arc-NPY neurons monosynaptically project to the LC (Yoon et al., 2013; Hwang and Lee, 2018). Two other feeding-related neuropeptides, glucagon-like peptide-1 receptor (Glp-1r) and proenkephalin-B (or prodynorphin; Pdyn), also play an important modulatory role on LC function (Kreibich et al., 2008; Rampersaud et al., 2012).

In the present experiment, we examined whether the LC-NE neural response and NPY-related genes were altered as a consequence of binge-like eating. For this, we used a previously described dietary-induced binge eating model, which employs repeated limited access to sweet-fat food (Bello et al., 2014a,b). This dietary-induced binge eating model also incorporates an intermittent caloric restriction element to mimic the reported influence calorie restriction has on binge eating in clinical populations (Haiman and Devlin, 1999; Roehrig et al., 2009; Pankevich et al., 2010; Rospond et al., 2015). Our previous findings indicated that the sensory-evoked LC-NE neural response was reduced in male rats *maintained* on intermittent calorie restriction and sweet-fat food access schedule for 10 weeks (Bello et al., 2014b). As females have a higher risk for developing an eating disorder (American Psychiatric Association, 2013), in the present study, we examined whether similar neural alterations were evident in female rats. One limitation of our previous studies using the dietary-induced binge eating model is that neural and behavioral outcomes measurement after a 6–10 week exposure (Bello et al., 2014a,b), which likely is more representative of the *maintenance* of the binge-like eating behavior. For this study, we determined whether these neural changes occurred after a shorter exposure (2.5 weeks) to the dietary protocol and after the binge-like eating phenotype was established. Additional feeding groups were included to determine the role of the binge-like eating and the contribution of the individual dietary variables (i.e., intermittent calorie restriction and sweet-food access) on LC neural responses and regional gene expression.

## Materials and Methods

### Animals

Fifty-two adult female Sprague Dawley (SD) rats (7–8 weeks of age) were acquired from ENVIGO (Frederick, MD), individually housed, and kept on a 12/12 h light-dark cycle. Rats were maintained on *ad libitum* standard chow (Purina Rat Diet 5012; 13% fat, 27% protein; 3.1 Kcal/g) unless specified otherwise, and water was available at all times. The animal care protocol was approved by the Institutional Animal Care and Use Committee of Rutgers University (OLAW #A3262-01, protocol #10-047) and complied with the NIH Guide for the Care and Use of Laboratory Animals.

### Feeding Schedules and Experimental Groups

The binge-like food used in this model of diet-induced binge eating is “sweetened fat” (8.6 kcal/g), comprised of vegetable shortening (Crisco, The J.M. Smucker Company, Orrville, OH) mixed with 10% sucrose (Domino Sugar; American Sugar Refining). One week prior to starting the respective feeding protocols, rats were exposed to a 24-h pre-exposure to the highly palatable sweetened fat. The pre-exposure was used to determine initial preferences for the binge food and to reduce novelty avoidance, and vaginal cytology was performed before and after pre-exposure to control for stage of estrous cycle. Rats were then randomized to one of four feeding schedules, with no initial differences in body weight or pre-exposure sweetened fat intake between groups. The four schedules included Restrict-Binge, Binge, Restrict, and Naïve groups, and each consisted of a 2.5-week feeding protocol. The *Restrict Binge* group had repeated cycles of intermittent 24-h calorie deprivation (beginning 1 h prior to lights off) followed by refeeding with standard chow and a concurrent 30-min access to sweetened fat. The exposure to intermittent calorie deprivation occurred on days 2 and 5, with the refeeding with standard chow and concurrent 30-min access to the sweetened fat on days 3 and 6 of the 7-day feeding schedule. In this fashion, the Restrict Binge group was exposed to a repeated cycle that consisted of three no restriction days (days 1, 4, and 7), two weekly episodes of calorie restriction (days 2 and 5), and two weekly episodes of scheduled refeeding with 30-min access to a highly palatable food (days 3 and 6). The second group, the *Binge* group, had *ad libitum* standard chow in addition to the 30-min access to the sweetened fat (days 3 and 6) at the same time and frequency as the Restrict Binge group. A third group, *Restrict* group, had an identical pattern of calorie deprivation with standard chow (days 2 and 5) as the Restrict Binge group, but did not have repeated access to the sweetened fat upon refeeding on days 3 and 6. A *Naive* group had ad libitum standard chow with no access to the sweetened fat, see Table 1 (Bello et al., 2014a,b). Rats were exposed to five binge cycles for a total of 2.5 weeks. Dietary-induced binge eating was operationally defined as the number of calories consumed in a 30-min period of time. Thus, the 2.5-week period was chosen in these studies because the Restrict Binge and Binge rats demonstrated a significant increase in caloric intake at the fifth access period compared with the first access period. Cumulative calorie intakes and body weights were measured every third day. Food intakes and body weights were measured to the nearest 0.1 g.

**Table 1:** Feeding groups for the dietary-induced binge eating protocol.

Groups	Calorie restriction (days 2 and 5)	Sweetened fat access (days 3 and 6)
Restrict Binge	Intermittent (24 h, twice a week)	Intermittent (30 min, twice a week)
Binge	None	Intermittent (30 min, twice a week)
Restrict	Intermittent (24 h, twice a week)	None
Naive	None	None

### Stage of the Estrous Cycle

As the stage of estrous has been associated with binge eating in humans and rodent models (Edler et al., 2007; Micioni Di Bonaventura et al., 2017), vaginal cytology was performed to determine the stage of estrous. On days 3 and 6 of the weekly schedule (i.e., refeeding days or “binge days”), vaginal cytology was performed 4 h before the schedule feeding. Briefly, the vaginal cavities of rats were lavaged with sterile saline (0.9%), and the cells were characterized by vaginal epithelial cell morphology. Proestrus/estrus was classified by the presence and relative number of nucleated epithelial and cornified cells. Metestrus/diestrus was classified by the presence and relative number of leukocytes (Goldman et al., 2007).

### Single-Unit Electrophysiology

After 2.5 weeks, and on day 2 (beginning week 3) of the feeding protocol, rats from each group were food deprived for 24 h. Single-unit electrophysiological recordings of LC neurons were performed under isoflurane anesthesia (Bello et al., 2014b). Glass electrodes were fabricated from micropipettes and filled with 0.5 M sodium acetate and 2% pontamine sky blue (impedance 4–7 MΩ). Single-unit recordings of LC spontaneous activity were recorded for at least 3 min, followed by evoked response to sciatic nerve stimulation (50 stimuli, 3.0 mA, 0.5 ms duration, 0.2 Hz) from 1 to 5 neurons per animal. Microiontophoresis of pontamine sky blue (15 μA for 10 min), followed by neutral red counterstain of 40 μm brain sections was used to confirm recording location in LC neurons. Only data from histologically confirmed LC neurons were included in the analysis. Peristimulus histograms (PISH) during sciatic nerve stimulation were constructed by synchronizing pulses to initiate 2-s sweeps. Sweeps began 500 ms prior to stimulus onset and concluded 1.5 s after the stimulus. The cumulative number of spikes during each 8-ms bin (250 bins total) was plotted. LC-NE neurons have a characteristic biphasic (excitatory-inhibitory) response following stimulation. Tonic discharge rate is represented by the 500 ms preceding the stimulus, and evoked discharge rate was defined as the post-stimulus firing that surpassed the mean tonic firing by two standard deviations. That is, the sensory evoked phase is represents by the 104 ms after stimulation, which is followed by a 392 ms inhibitory phase (Bello et al., 2014b).

### RNA Extraction and Quantitative Real-Time Polymerase Chain Reaction

RNA isolation and two-step quantitative real-time polymerase chain reaction (RT-qPCR) gene expression analysis were performed in the Arc of the hypothalamus and LC of the pons in female SD rats exposed to Restrict-Binge, Binge, Restrict, and Naive following the completion of the binge protocol for 2.5 weeks. Similar to the terminal experimental conditions of the single-unit recording, all groups were food deprived for 24 h prior to euthanasia. Animals underwent live decapitation, and brains were sectioned into 1-mm coronal slices using a brain matrix (Ted Pella) to include the Arc (−1.80 to −2.16 mm from Bregma) and LC (−9.48 to −9.96 mm from Bregma) regions (Paxinos and Watson, 2007). The tissue slices were then transferred to RNAlater (Life Technologies) and stored overnight at 4°C. The following day, Arc and LC nuclei were extracted *via* microdissection, and tissue was stored in 1.5 ml nuclease-free pestle microtubes, at −80°C. Total Arc and LC RNA was isolated using Ambion RNAqueous-Micro kits (Life Technologies, Inc.), and treated with deoxyribonuclease I at 37°C for 30 min to minimize contamination by genomic DNA. RNA integrity was analyzed using a NanoDrop ND-2000 spectrophotometer (ThermoFisher). Superscript III reverse transcriptase (Life Technologies), 4 μl 5× buffer, 25 mM MgCl_2_, 10 mM deoxynucleotide triphosphate (CLONTECH Laboratories), 100 ng random hexamer primers (Promega Corp.), 40 U/μl Rnasin (Promega), and 100 mM dithiothreitol in diethylpyrocarbonate-treated water (Gene Mate; Bioexpress) were used to synthesize cDNA from 200 ng of total RNA, in a total volume of 20 μl. Reverse transcription was performed using the following thermocycler protocol: 5 min at 25°C, 60 min at 50°C, and 15 min at 70°C. The cDNA was diluted 1:20 using nuclease-free water (Gene Mate; Bioexpress) for a final cDNA concentration of 0.5 ng/μl and stored at 20°C. Male rat basal hypothalamus test tissue RNA was used as positive and negative controls (no reverse transcriptase) and processed simultaneously with the experimental samples (Gotthardt et al., 2016). Tissues that failed to produce sufficient RNA or CT values were excluded from the data set (*n* = 1 for LC from Restrict group and *n* = 1 from Arc from Binge group). The following primer sets and Gen Bank accession numbers were used for qPCR. For rat neuropeptide Y (*Npy*), F: 5-CTCTGCGACACTACATCAATCTC-3 and R: 5-GCTGGATCTCTTGCCATATCTC-3 (NM_012614.2); NPY Y1 receptor (*Npy1r*), F: 5-GGCTGTCTTACACGACTCTTCT-3 and R: 5-TGGTCTCACTGGACCTGTACTT-3 (NM_001113357.1); NPY Y5 receptor (*Npy5r*), F: 5-GGGCATCCCGAGGATTT-3 and R: 5-CCGAGCAGCAGCTGTAT-3 (NM_012869.1); glucagon-like peptide-1 receptor (*Glp-1r*), F: 5-GCCCTCAAGTGGATGTAT-3 and R: 5-ACCAGCAACCAGTAGTAG-3 (NM_012728.1); glyceraldehyde-3-phosphate dehydrogenase (*Gapdh*), F: 5-GTGATGCTGGTGCTGAGTA-3 and R: 5-GCGGAGATGATGACCCTTT-3 (NM_017008.4). The following amplicon context sequences and unique assay IDs from SYBR® Green PrimePCR Assays (BioRad) were used for qPCR. For rat proenkephalin-B preproprotein (*Pdyn*), 5-GATTTGGTAGCCTTCAAGGCTTCCTCTGTGGCACTTCTCTGAGCTAGCGTCAGG GCTCCTTCTGAATCTTGGATCGGCCATCCTATCACCTGATCAGCCAGAAGC-3 (ID: qRnoCED0003526); for neuropeptide Y receptor type 2 (*Npy2r*), 5-AATAAAAAAGTTGGTTACTGTGCGCATGCTCTTGAATTTGATCACCACATGGATTA CCAGAGAGTTGCCAACTACGCCCAGCAAGATGATGGAACAATAGGCCAATATAA GGAC-3 (ID: qRnoCED0004910). For qPCR, 4 μl of cDNA template (an equivalent of 2 ng total RNA) was amplified using Sso Advanced SYBR Green (Bio-Rad Laboratories, Inc.) on a CFX-Connect real-time PCR instrument (Bio-Rad Laboratories). Standard curves for each primer pair were prepared using serial dilutions of basal hypothalamus cDNA in duplicate to determine the efficiency of each primer pair. All efficiencies expressed as percent efficiency were approximately equal (one doubling per cycle, 90–100%). The amplification protocol for all the genes was as follows: initial denaturing at 95°C for 3 min followed by 40 cycles of amplification at 94°C for 10 s (denaturing), 60°C for 45 s (annealing), and completed with a dissociation step for melting point analysis with 60 cycles of 95°C for 10 s, 65–95°C (in increments of 0.5°C) for 5 s, and 95°C for 5 s. The relative mRNA expression data were analyzed using the cycle threshold (CT) method. The reference gene was glyceraldehyde-3-phosphate dehydrogenase (*Gapdh*). Positive and negative controls were added to each amplification run and included a water blank. Quantification values were generated only from samples showing a single product at the expected melting point. Final relative quantitation was done using the comparative CT method. The data were reported as relative mRNA expression. To determine the CT for each transcript, the threshold was consistently set at the lowest point of the exponential curve where the slope of the curve was the steepest for all plates. The relative linear quantity of target molecules was calculated using the formula 2^−δδCT^. All gene expression data were expressed as an *n*-fold difference relative to the Naive group (Gupta et al., 2018).

### Statistical Analyses

Food intake and body weight were analyzed by repeated measures ANOVA. Spontaneous, tonic, and evoked rates (Hz), as well as signal-to-noise, were separately analyzed with ANOVA to determine differences among groups and two-way ANOVA for individual dietary variables (i.e., intermittent calorie restriction and sweet-food access). PISH data were analyzed by a repeated-measures ANOVA for group difference and a multivariate ANOVA for individual dietary variables. Relative gene expression levels were analyzed using ANOVA for group differences and two-way ANOVA for individual dietary variables. *Post hoc* Newman-Keuls tests were performed unless otherwise specified. All statistical analyses were performed using Statistica 7.1 software (StatSoft, Tulsa, OK, USA) and significance was set at *α* = 0.05.

## Results

### Feeding Behavior, Bodyweight, and Estrous Cycle

**Figure 1:**
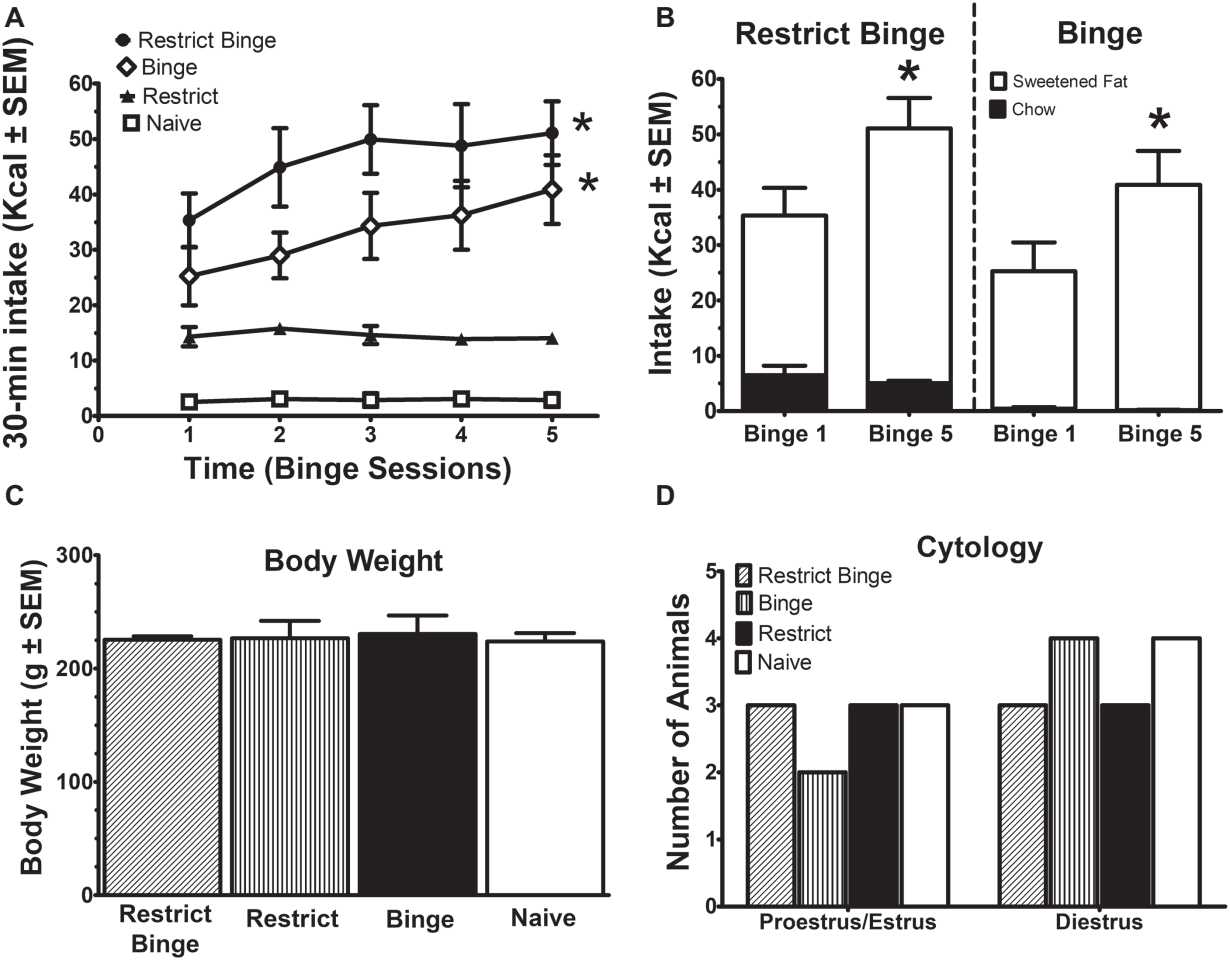
Intermittent limited access to sweet food promotes dietary-induced binge eating in female rats. Data are expressed as mean ± SEM. Rats were exposed to intermittent 30-min eating bouts twice a week. Feeding groups are illustrated in Table 1. The Restrict Binge group (*n* = 6) received intermittent calorie deprivation (24 h) and 30-min access to “sweetened fat” (vegetable shortening +10% sucrose; 8.6 kcal/g), the Binge group (*n* = 6) received intermittent 30-min access to sweetened fat, the Restrict group (*n* = 6) received intermittent calorie deprivation (24 h), and the Naive group (*n* = 7) received neither sweetened fat access nor intermittent calorie deprivation. **(A)** Intakes (kcal) during the 30-min access period on day 3 and day 6. For both the Restrict Binge and Binge groups, * indicates *p* < 0.05 from first 30-min access period (i.e., binge) to the last 30-min access period for the Restrict Binge and Binge groups. **(B)** Comparison of the total binge and last binge period intake in the Restrict Binge and Binge groups. Sweetened fat intake is represented by the white bars, whereas the black bars are chow intake. For both the Restrict Binge and Binge groups, * indicates *p* < 0.05 for sweetened fat intake from binge 1 and binge 5. **(C)** Final body weights at the completion of 2.5 weeks. **(D)** Vaginal cytology on the electrophysiological recording day.

Binge-like eating was defined as number of calories in a relatively short period of time and increased intake over time. The binge eating was defined by the difference among the groups as result of the dietary conditions (see Table 1). The Restrict Binge and Binge had greater caloric intakes during the twice weekly 30 min access period and demonstrated an escalation in sweetened fat over the 2.5 weeks compared with Restrict and Naive groups. There was an effect over time for the Restrict Binge [*F* (4, 20) = 3.1, *p* < 0.05] and Binge [*F* (4, 20) = 3.7, *p* < 0.05] groups. *Post hoc* testing indicated the 30-min total Kcal intake for Binge 5 was greater than Binge 1 for both groups (*p* < 0.05; Figure 1A). In addition, the intake of the sweetened fat increased from Binge 1 to Binge 5 (*p* < 0.05). There were no differences in standard chow intake from Binge 1 to Binge 5 (Figure 1B). There were no differences in body weights at the completion of the 2.5 week feeding protocol, Figure 1C. Over the 2.5 weeks, there were no differences in stage of estrous cycle among the feeding groups. The stage of estrous on the recording day is illustrated in Figure 1D.

### Single-Unit Electrophysiological Recording of Locus Coeruleus Neurons

**Figure 2:**
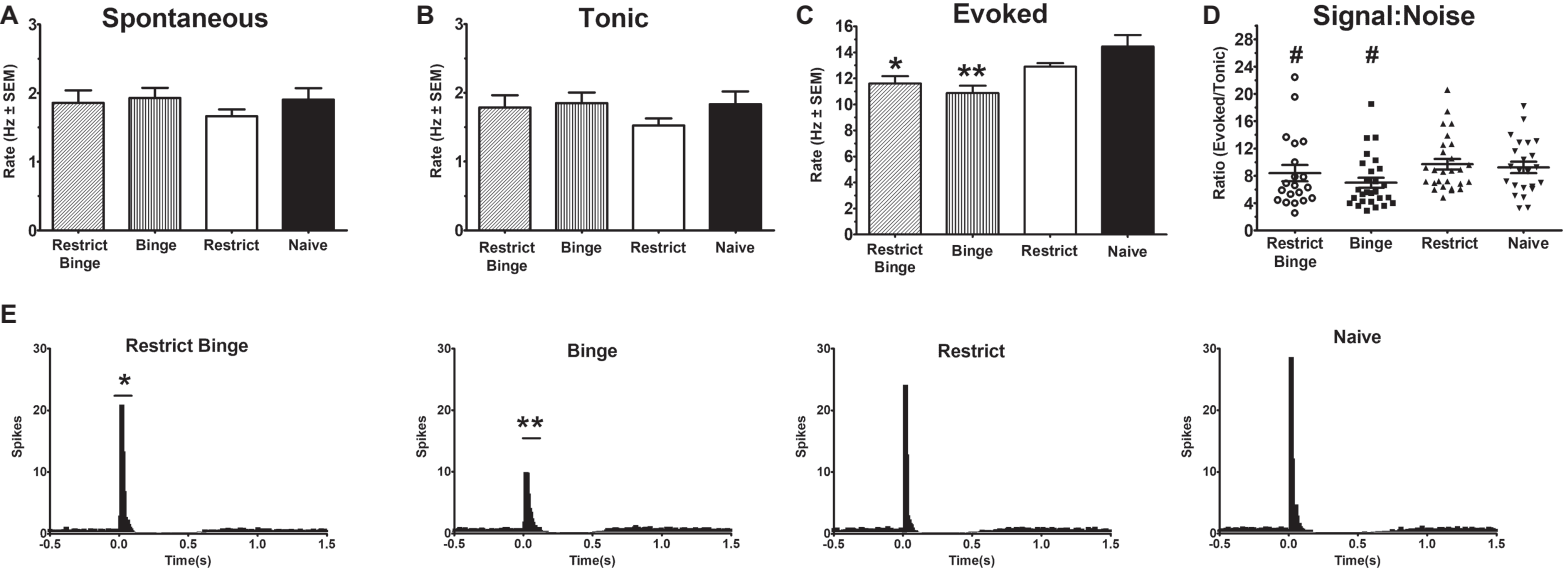
Dietary-induced binge eating reduces sensory-evoked neural activity of the locus coeruleus neurons. Single-unit electrophysiological recordings of LC neurons were performed under isoflurane anesthesia. Sensory evoked activity was sciatic nerve stimulation (50 stimulus trains, 3.0 mA, 0.5 ms duration; each stimulus train were 2 s in total duration). Recording occurred on *Day 3* of the feeding protocol following a 24 h calorie deprivation for each group: Restrict Binge (20 cells from six rats), and Binge (27 cells from six rats), Restrict (22 cells from six rats), and Naive (22 cells from seven rats). Rates are reported in hertz (Hz) ± SEM. **(A)** Spontaneous (3 min prior to stimulus train). **(B)** Tonic (500 ms preceding stimulus in the stimulus train) **(C)** Evoked rate (104 ms following sensory stimulus); for the Restrict Binge group, * indicates *p* < 0.05 from Naive group; for the Binge group, ** indicates *p* < 0.005 from Naive and Restrict groups. **(D)** Signal-to-noise ratio was reduced in rats receiving sweetened fat exposure (*p* < 0.05; indicated as #). **(E)** Cumulative peristimulus histogram (PISH) data demonstrate there was only a significant difference in the evoked phase (104 ms following stimulus onset); for the Restrict Binge group, * indicates *p* < 0.05 from Naive group, for the Binge group, ** indicates *p* < 0.005 from Naive and Restrict groups.

Neural activity was recorded from Naive (22 cells from seven rats), Restrict (22 cells from six rats), Restrict Binge (20 cells from six rats), and Binge (27 cells from six rats). There were not any differences in spontaneous or tonic activity; see Figures 2A,B. However, sensory evoked activity was different among the groups [*F* (3, 93) = 7.1, *p* < 0.005]. *Post hoc* testing revealed the Restrict Binge had a lower sensory evoked rate compared with the Naive group (*p* < 0.05), whereas Binge had a reduced rate compared with the Naive and Restrict groups (*p* < 0.005); see Figure 2C. When expressed as a ratio of evoked/tonic activity (i.e., signal-to-noise) there were not differences among the groups [*F* (3, 93) = 2.06, *p* = 0.10]; see Figure 2D. The PISH data revealed a similar pattern, such there was an overall group effect for the evoked phase [*F* (3, 92) = 7.5, *p* < 0.005]. There was also a group X time effect [*F* (39, 1,196) = 4.7, *p* < 0.005]. *Post hoc* testing revealed that the Restrict Binge group had a reduced evoked phase compared with Naive group (*p* < 0.05). The Binge group had a reduced evoked phase compared with Naive and Restrict groups (*p* < 0.005 for both); see Figure 2E. When analyzed for the dietary variables, there were reduced LC sensory evoked rates in rats with repeated exposed to the sweetened fat condition [*F* (1, 93) = 16.76, *p* < 0.0001]. There was also a reduced signal-to-noise in rats with repeated sweetened fat exposure [*F* (1, 93) = 4.11, *p* < 0.05]. For the dietary variables, the evoked phase of the PISH demonstrated a reduced response with repeated sweetened fat exposure [*F* (1, 92) = 18.14, *p* < 0.005] with a sweetened fat exposure × time [*F* (13, 1,196) = 3.29, *p* < 0.0005].

### Relative Gene Expression in the Pontine Locus Coeruleus and Hypothalamic Arcuate Nucleus Regions

**Figure 3:**
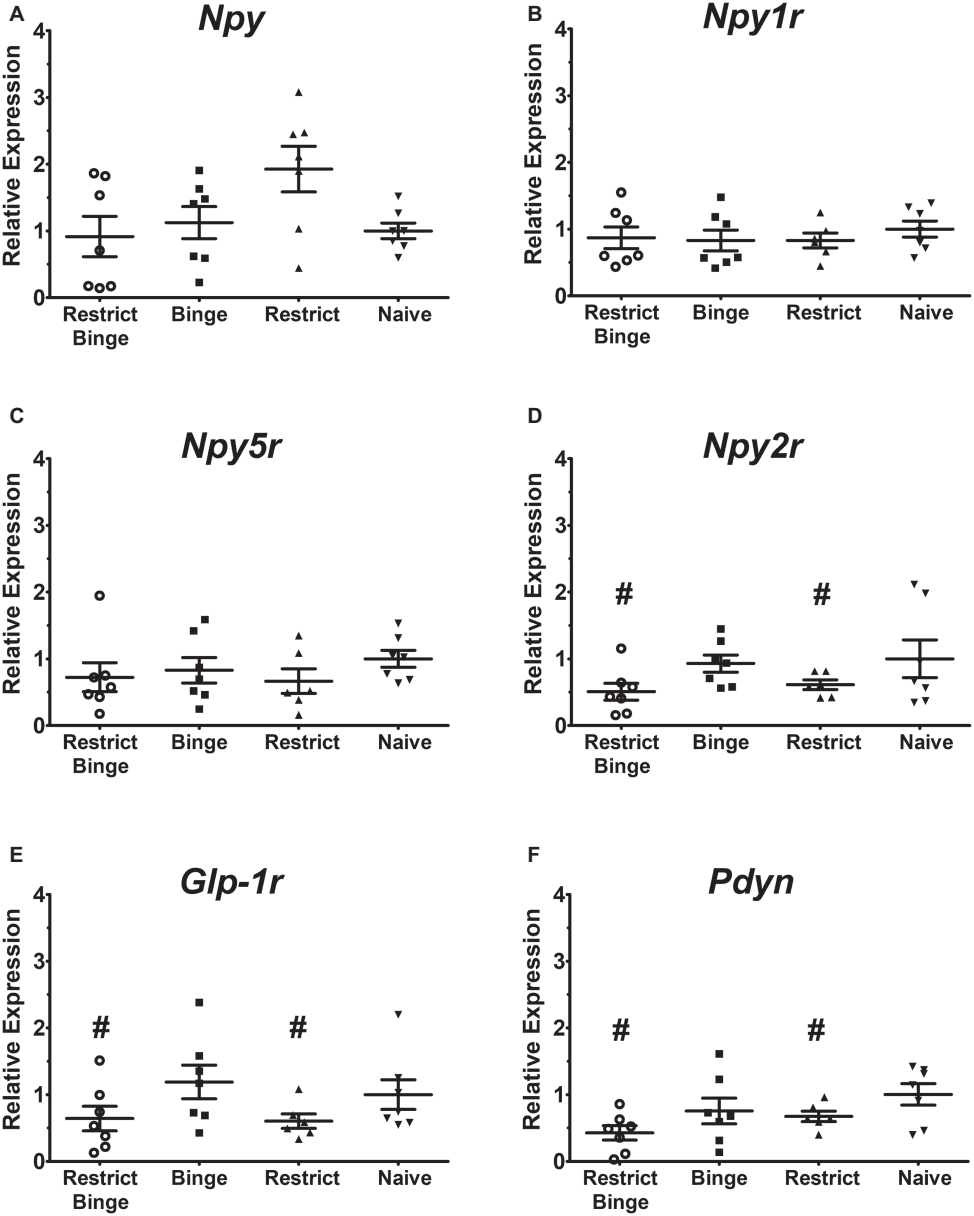
Relative gene expression in the pontine locus coeruleus region of female rats after 2.5 weeks on the dietary-induced binge eating protocol. Data were expressed as relative expression calculated using δδ cycle threshold (CT) method and as a *n*-fold difference relative to the mean of the Naive group. All gene expression data were expressed as an *n*-fold difference relative to the Naive group. **(A)** neuropeptide Y (*Npy*), **(B)** NPY Y1 receptor (*Npy1r*), **(C)** NPY Y5 receptor (*Npy5r*), **(D)** NPY Y2 receptor (*Npy2r*), **(E)** glucagon-like peptide-1 receptor (*Glp-1r*), **(F)** proenkephalin-B preproprotein (*Pdyn*). Gene expression for *Glp-1r*, *Npy2r*, and *Pdyn* were reduced with repeated intermittent restriction (*p* < 0.05 for all; indicated as #). All groups were *n* = 7, except Restrict (*n* = 6). Mean ± SEM.

**Figure 4:**
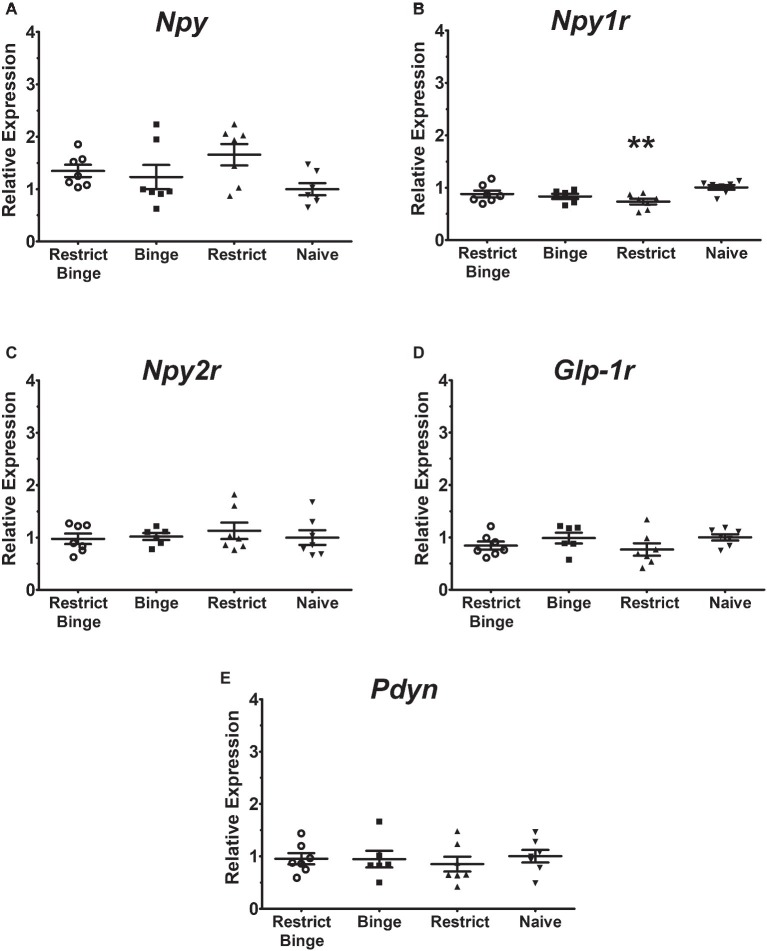
Relative gene expression in the hypothalamic arcuate nucleus region of female rats after 2.5 weeks on the dietary-induced binge eating protocol. Data were expressed as relative expression calculated using δδ cycle threshold (CT) method and as a *n*-fold difference relative to the mean of the Naive group. All gene expression data were expressed as an *n*-fold difference relative to the Naive group. **(A)** neuropeptide Y (*Npy*), **(B)** NPY Y1 receptor (*Npy1r*), **(C)** NPY Y2 receptor (*Npy2r*), **(D)** glucagon-like peptide-1 receptor (*Glp-1r*), **(E)** proenkephalin-B preproprotein (*Pdyn*). All groups were *n* = 7, except Binge (*n* = 6). Mean ± SEM. For the Restrict group, ** indicates (*p* < 0.01) from Naive group for *Npy1r* expression.

In the pontine locus coeruleus region, there were no gene expression differences in *Npy*, *Npy1r*, *Npy5r*, *Npy2r*, and *Glp-1r* (Figures 3A–E). The *Pdyn* expression did approach significance [*F* (3, 23) = 2.70, *p* = 0.069] with the Restrict Binge and Restrict groups trending lower than the Naive group (Figure 3F). When analyzed for the individual dietary variables, there were reductions in the expression of *Glp-1r* [*F* (1, 23) = 5.3, *p* < 0.05], *Npy2r* [*F* (1, 23) = 5.2, *p* < 0.05], and *Pdyn* [*F* (1, 23) = 5.0, *p* < 0.05] with repeated intermittent restriction. In the arcuate nucleus region (Figures 4A–E), *Npy1r* expression was different among groups [*F* (3, 23) = 4.51, *p* < 0.05]. *Post hoc* testing revealed that there was 26.7% lower expression of *Npy1r* in the Restrict group compared with Naive (*p* < 0.01) (Figure 4B). When analyzed for the individual dietary variables, there was a reduction in the expression of *Npy1r* with the repeated intermittent restriction [*F* (1, 23) = 4.4, *p* < 0.05] and sweetened fat exposure × restriction [*F* (1, 23) = 8.6, *p* < 0.01]. *Post hoc* testing indicated the no sweetened fat/restriction condition (i.e., Restrict group) had reduced expression compared with the no sweetened fat/no restriction condition (i.e., Naive group; *p* < 0.05).

## Discussion

The first objective of this study was to characterize sensory-evoked neural activity in LC-NE neurons *in vivo* in young adult female rats displaying a binge eating phenotype. Our findings indicated that female rats exposed to twice weekly intermittent 30-min access to sweetened fat displayed an overeating response that increased over the 2.5 weeks period. The binge-like eating phenotype was displayed in both Restrict Binge and Binge groups, suggesting that the phenotype was not influenced by the intermittent calorie restriction prior to the sweetened fat access. In both the Restrict Binge and Binge groups we observed reduced sensory-evoked LC neural activity compared with the Naive group. In addition, the Binge group had reduced evoked LC neural activity compared with the Restrict group (intermittent calorie restriction only). Analysis for the individual dietary factors indicated that the repeated sweetened fat access, not the dietary restriction, reduced LC sensory-evoked activity. The repeated sweetened fat access condition also decreased the signal-to-noise ratio. This suggest the repeated highly palatable food access dampens the neural activation to stress, as has been demonstrated by others (Christiansen et al., 2011; Liang et al., 2013; Sharma et al., 2013). We previously reported that male rats maintained on the Restrict Binge feeding protocol for 10 weeks (additional 7.5 weeks) had a reduced sensory-evoked LC neural response (Bello et al., 2014b). Taken together, the findings of these two studies suggest that sensory evoked LC-NE neural response, not spontaneous activity, is attenuated as consequence of dietary-induced binge eating. One speculation is that the reduced sensory evoked activity in the NE-LC system as a consequence of repeated access binge food could have implications in reducing the elevated negative affect associated with binge eating in clinical populations (Haedt-Matt and Keel, 2011). Importantly, on the recording day, we did not observe differences in estrous cycle stage across animals, indicating that group differences in evoked LC-NE responses were not a result of differential cycle stage. In addition, all estrous stages were equally observed across all groups, indicating that normal cycling was not affected by the feeding paradigms. Given that ovarian steroids are known to modulate the activity of LC neurons in females (Szawka et al., 2009), it will be important that future studies investigate whether cycle stage is associated with the magnitude of LC-NE suppression following the binge-like eating behavior.

One limitation of the present study is that we did not examine whether the threshold for evoking LC activity was altered as a consequence of the dietary-induced binge eating. Previous studies have indicated that nociceptive signaling is enhanced by prolonged calorie deprivation (48 h) in female rats (Khasar et al., 2003), but the influence of intermittent bouts of calorie deprivation is not clear. In BN women, pain thresholds increased immediately (<1 h) after a binge/vomiting episode, but pain thresholds were altered from baseline following overnight fast (Raymond et al., 1999). Elevated pain detection sensitivity also has been reported in obese BED subjects compared with non-obese and obese controls (Raymond et al., 1995). To minimize any acute influence on the feeding schedule on the electrophysiology recordings, all rats were exposed to a 24 h calorie restriction beforehand. However, we have not determined the long-term influence of the dietary-induced binge eating protocol on nociceptive outcomes; this will be important for future studies to interrogate.

The second objective of our study was to determine the influence of the dietary-induced binge eating on the gene expression of NPY and related markers in the Arc and LC regions. Notably, we did observe a decrease in Arc *Npy1r* expression in the Restrict group, compared with Naive group. Previous studies have indicated that a single bout of calorie restriction (48 h) in rats reduces the expression of Arc *Npy1r* mRNA and Y1 immunoreactivity (Cheng et al., 1998). In our study, all feeding groups had similar body weights and were exposed to a 24 h calorie deprivation prior to harvesting the tissue for gene expression analysis, indicating that the reductions in *Npy1r* gene expression observed here may have been linked to the intermittent bouts of calorie restrictions conditions of the Restrict feeding group. Since the Restrict Binge group had similar intermittent bouts of calorie restriction, one possibility is that the sweetened fat intake by the Restrict Binge normalized the gene expression of *Npy1r*. It is also interesting to note that a previous study identified a link between a prepro-NPY single nucleotide polymorphism (Leu7Pro) and NE plasma levels (Jaakkola et al., 2005). However, a follow-up study showed that this particular polymorphism was not associated with either BN or BED (Kindler et al., 2011), indicating that NPY-driven changes in peripheral NE levels may not be critically involved in these pathologies. One limitation of our study is that we did not examine the functional connection between Arc *Npy1r* signaling and LC neural activity, and thus, the directionality of any influence between these systems remains unclear. Further studies will be required to fully characterize the relationship between NPY and central NE signaling following binge-like eating.

Besides NPY and receptor related genes, we also examined the expression of two other feeding-associated genes, *Glp-1r* and *Pdyn*. We examined *Glp-1r* gene expression as Glp-1r mRNA is distributed in LC and central GLP-1 signaling plays a key role in modulating feeding behaviors (Merchenthaler et al., 1999; Moran, 2009; Rinaman, 2010). We examined *Pdyn*, as there is evidence that dynorphin-containing neurons directly innervate NE-containing cells of the LC (Reyes et al., 2007), and kappa receptor agonists have been demonstrated to selective reduce sensory-evoked LC neural activity and not influence spontaneous or tonic LC activity (as we observed here following binge experience) (Kreibich et al., 2008). When examined for the individual conditions, *Glp1r* and *Pdyn* were reduced in the groups that experienced repeated intermittent caloric restriction in the LC regions. Neither *Glp-1r* nor *Pdyn* were differential expressed in the Arc region. This indicates that changes in GLP-1 and dynorphin signaling in the LC could represent compensatory changes following repeated bouts of intermittent caloric restriction.

## Conclusions

Early studies suggest a dysregulation of central NE associated with recurrent binge eating episodes (Kaye et al., 1990a,b; El-Giamal et al., 2000; Fassino et al., 2004; Silveira et al., 2005; McElroy et al., 2007). We show that young female rats exposed to repeated bouts of limited access to a sweet-fat food decreased the sensory-evoked activity LC neurons. These findings parallel those observed previously in male rats following an extended history of dietary-induced binge-like eating, and future studies directly comparing the time course of changes in LC-NE function in males compared with females may add to our understanding of differential susceptibility to eating disorders between sexes in clinical populations. Of note, changes in LC-NE function following binge experience were associated with differential expression of several feeding-related genes in either Arc or LC, pointing to the potential involvement of other neuropeptides important for modulation of LC-NE function. The functional implications of Arc NPY1 receptor signaling following repeated intermittent caloric restriction requires further investigation. Together, our data indicate that dietary-induced binge eating alters the neural response of LC neurons to sensory stimuli and dampen the neural stress response, and thus might be an important mechanism involved in stress-driven binge-eating pathology.

## Data Availability

The datasets generated for this study are available on request to the corresponding author.

## Ethics Statement

This study was carried out in accordance with the recommendations of NIH Guide for the Care and Use of Laboratory Animals. The protocol was approved by the Institutional Animal Care and Use Committee of Rutgers University (OLAW #A3262-01, protocol #10-047).

## Author Contributions

NB conceived and designed the study, analyzed the data, and wrote and edited the manuscript. C-YY conducted the experiment and wrote part of the manuscript. MJ analyzed the data and edited the manuscript.

### Conflict of Interest Statement

The authors declare that the research was conducted in the absence of any commercial or financial relationships that could be construed as a potential conflict of interest.
